# A novel multikinase inhibitor SKLB‐YTH‐60 ameliorates inflammation and fibrosis in bleomycin‐induced lung fibrosis mouse models

**DOI:** 10.1111/cpr.13081

**Published:** 2021-06-14

**Authors:** Hongyao Liu, Xiuli Wu, Cailing Gan, Liqun Wang, Guan Wang, Lin Yue, Zhihao Liu, Wei Wei, Xingping Su, Qianyu Zhang, Zui Tan, Yuqin Yao, Liang Ouyang, Luoting Yu, Tinghong Ye

**Affiliations:** ^1^ Sichuan University‐Oxford University Huaxi Gastrointestinal Cancer Centre State Key Laboratory of Biotherapy, West China Hospital Sichuan University Chengdu China; ^2^ West China School of Public Health and Heathy Food Evaluation Research Center and West China Fourth Hospital Sichuan University Chengdu China

**Keywords:** YTH‐60, multikinase inhibitor, pulmonary fibrosis, immune cells, epithelial‐mesenchymal transition

## Abstract

**Objectives:**

Idiopathic pulmonary fibrosis (IPF) is marked by the excessive accumulation of extracellular matrix, which participates in a variety of chronic diseases or injuries and seriously threatens human health. Due to the side effects of clinical drugs, there is still a need to develop novel and less toxic drugs to treat pulmonary fibrosis.

**Materials and Methods:**

SKLB‐YTH‐60 was developed through computer‐aided drug design, de novo synthesis and high‐throughput screening. We employed the bleomycin (BLM)‐induced lung fibrosis animal models and used TGF‐β_1_ to induce the epithelial‐mesenchymal transition (EMT) of A549 cells in vitro. Meanwhile, the protein expression of collagen I and the α‐smooth muscle actin (α‐SMA), E‐cadherin, p‐FGFR1, p‐PLCγ, p‐Smad2/3 and p‐Erk1/2 was detected by western blot.

**Results:**

YTH‐60 has obvious anti‐proliferative activity on fibroblasts and A549 cells. Moreover, YTH‐60 could impair the EMT of A549 cells and suppressed fibrosis by inhibiting FGFR and TGF‐β/Smad‐dependent pathways. Intraperitoneal administration of preventive YTH‐60 could significantly reduce the degree of fibrosis in mice and regulate the imbalance of the immune microenvironment. In addition, we observed that therapeutic YTH‐60 treatment attenuated fibrotic changes in mice during the period of fibrosis. Importantly, YTH‐60 has shown an acceptable oral bioavailability (*F* = 17.86%) and appropriate eliminated half‐life time (*T*_1/2_ = 8.03 hours).

**Conclusions:**

Taken together, these preclinical evaluations suggested that YTH‐60 could be a promising drug candidate for treating IPF.

## Graphical abstract

Schematic model for anti‐fibrotic activity of SKLB‐YTH‐60. In vitro YTH‐60 inhibits the activation of fibroblasts and the EMT of epithelial cells induced by TGFβ1. Intraperitoneal injection of YTH‐60 not only inhibits the bleomycin‐induced lung inflammation, regulates the lung immune microenvironment, but also prevents and reverses pulmonary fibrosis. Finally, the anti‐fibrosis effect of YTH‐60 may be via inhibiting of FGFR and TGF‐β/Smad signaling pathway.

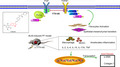

## 1 INTRODUCTION

Idiopathic pulmonary fibrosis (IPF) is a chronic and lethal lung disease associated with fibroblast activation, myoblast proliferation and extracellular matrix (ECM) deposition.^1^ Although the mechanism is unclear, most studies have shown that the increased proliferation of fibroblasts is the most direct cause of fibrosis.^2^ Currently, the complex aetiology and the not accurate diagnosis of pulmonary fibrosis, leading to the treatment progress is slow. Gene therapy is a potential treatment; however, successfully applying it in clinical practice has been a formidable challenge.^3^, ^4^ Only two novel anti‐fibrotic therapies approved by the FDA, a pyridinone derivative pirfenidone and a multi‐target tyrosine kinase inhibitor Nintedanib.^5^, ^6^ However, due to adverse reactions and limited efficacy of current drugs, it is still far from satisfaction in protecting against pulmonary fibrosis.^7^, ^8^ Other target inhibitors of TGF‐β‐signalling that are currently being evaluated in clinical trials such as fresolimumab (GC‐1008) and thalidomide.^9^ There is still a need for developing new and less toxic drugs to treat pulmonary fibrosis. Therefore, our research group has been interested in the design, synthesis and biological evaluation of novel multi‐kinase inhibitors as potential new anti‐fibrosis agents.

Myofibroblasts are generally considered to be important effector cells in the development of fibrosis,^10^ which are the main source of collagen I and the characteristic is expression of α‐smooth muscle actin (α‐SMA). Many mediators and growth factors have been involved in the process of lung fibrosis.^11^, ^12^ Among these molecules, the transforming growth factor β 1(TGF‐β_1_) is an important profibrotic factor in lung fibrosis and wound healing can markedly cause the differentiation of fibroblasts into myofibroblasts and induces EMT in alveolar epithelial cells (AECs).^13^ TGF‐β_1_ can elevate the expression of fibrosis marker α‐SMA which promoter harbours Smad binding elements, which specifically bind Smad3.^14^ In addition, Studies have shown that the FGFR signalling pathway is involved in the pathogenesis of IPF and cooperatively cross‐talks with TGF‐β_1_.^15^ After the two receptor molecules form a dimer on the membrane, the tails of the intracellular domains contact each other to activate their protein kinases to autophosphorylate the tyrosine residues in the tails, leading to the activation of downstream signals Erk and PLCγ. The FGFR signalling pathway takes an important part in tissue repair, embryogenesis and wound healing.^16^ Furthermore, multiple studies have shown that TGF‐β_1_ could change the sensitivity of FGFR in human primary lung fibroblasts.^17^, ^18^ Much of the research indicated that FGF‐2 and FGFR1IIIc are involved in EMT.^19^ Antibodies that neutralize FGF2 successfully inhibited the fibrosis process mediated by TGF‐β_1_.^15^ In short, FGF pathway could be an ideal target for anti‐fibrotic therapeutic approaches.

Our goal was to develop a new multi‐kinase inhibitor that could potently block the FGF/FGFR signalling cascade with low toxicity. Considering the anti‐fibrosis potential of tyrosine kinase inhibitor, we independently developed a series of compounds. Among these compounds, YTH‐60 showed outstanding kinase inhibitory activity, including FGFR1, FGFR2, FGFR3, Abl and VEGFRs. This compound has also shown anti‐proliferative activities against a panel of fibroblast cells. In this study, we used the clinical drug Nintedanib as a positive control and evaluated the activity of YTH‐60 in inhibiting lung fibrosis in vitro and in vivo. Moreover, further mechanistic studies revealed that YTH‐60 ameliorated fibrosis by inhibiting both FGFR and TGFβ/Smad‐dependent pathways. Taken together, these preclinical evaluations suggest that YTH‐60 could be a promising drug candidate for the treatment of fibrotic diseases.

## 2 MATERIALS AND METHODS

### 2.1 Synthesis and preparation of the compound YTH‐60

**FIGURE 1:**
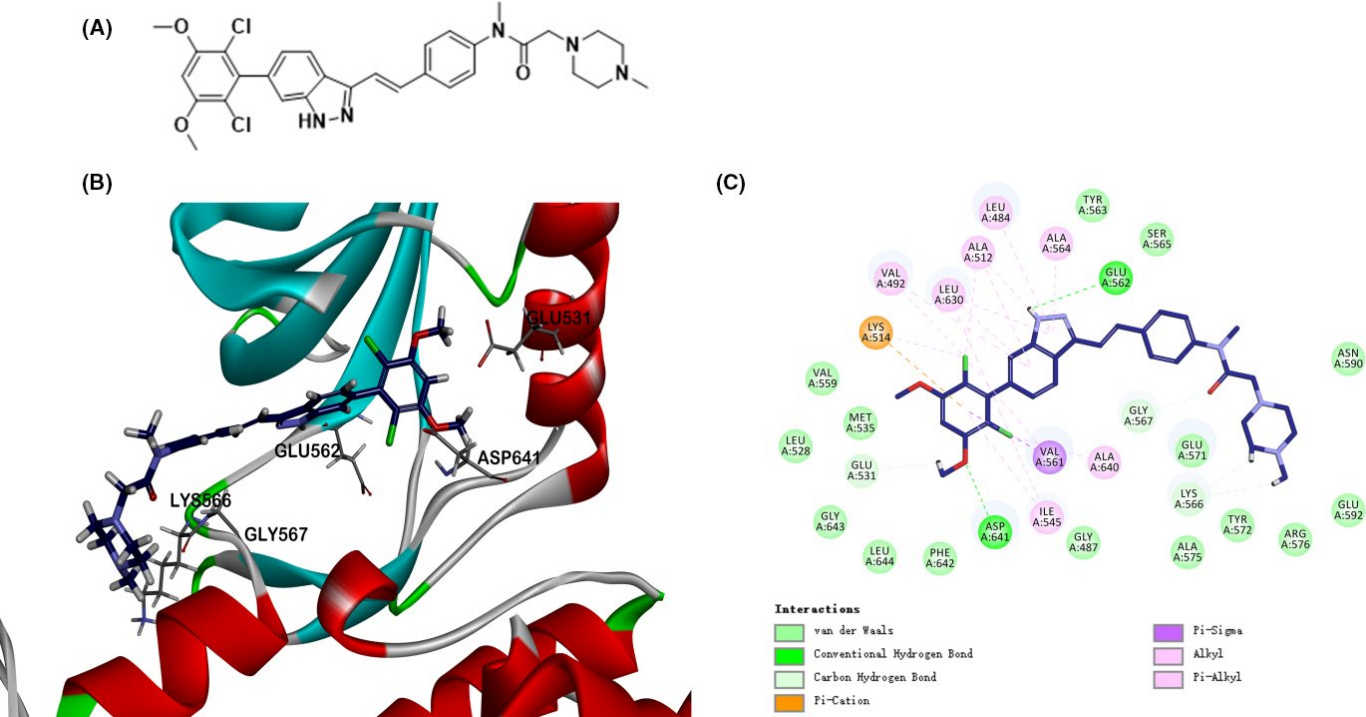
A, Chemical structure of YTH‐60. B, Docking of YTH‐60 into FGFR1 kinase X‐ray crystal structure in the three‐dimensional structure (PDB ID:5UR1). C, A two‐dimensional interaction map of YTH‐60 and FGFR1

YTH‐60(*E*)‐*N*‐(4‐(2‐(6‐(2,6‐dichloro‐3,5‐dimethoxyphenyl)‐1*H*‐indazol‐3‐yl) vinyl) phenyl)‐*N*‐methyl‐2‐(4‐methylpiperazin‐1‐yl) acetamide was synthesized by our team and the structural formula is shown in Figure 1A and Figure S1. YTH‐60 was dissolved in DMSO and the stock solution diluted to the appropriate concentration for all in vitro studies. The final concentration of DMSO was about 0.1% (v/v^−1^). For in vivo animal experiments, YTH‐60 was dissolved in DMSO, PEG400 and Normal saline (0.5:4.5:5). ^1^H NMR and ^13^C NMR spectra were recorded on a Bruker Avance‐400 spectrometer or Varian spectrometer with tetramethylsilane (TMS) as an internal standard. The purities of all final compounds were greater than 95%.

### 2.2 Drugs and regents

Cell count kit‐8 (CCK‐8) was purchased from MedChemExpress. MTT and DMSO were purchased from Sigma. TGF‐β_1_ and FGF‐basic were purchased from Novoprotein. PEG 400 was purchased from Sigma. Collagenase Type IV was purchased from GIBCO. Bleomycin sulphate was purchased from Chengdu Synguider Technology Co., Ltd. Nintedanib were purchased from Chengdu Giant Pharmaceutical Technology Co., Ltd (C_31_H_33_N_5_O_4_, MW: 539.62; CAS#656247‐17‐5). Antibodies against p‐Smad2/3 (Cat #8828S) and Smad2/3 (Cat #8685S) were purchased from Cell Signaling Technology. α‐SMA (Cat #ab5694), collagen I (Cat#ab88147), ERK1/2 (Cat #ab32537), FGFR1 (Cat #ab824), p‐FGFR1 (Cat #ab59194), PLCγ (Cat #ab76155), p‐PLCγ (Cat #D25A9) and β‐actin (Cat #ab8226) were purchased from Abcam. GAPDH (Cat #18AF0404, ZSJQ‐BIO). The dilution ratio of all primary antibodies is 1:1000, and the dilution ratio of secondary antibodies is 1:3000.

### 2.3 Molecular modelling

The molecular docking studies were accomplished by using GOLD 5.0. The crystal structure of FGFR1 (PDB ID: 5UR1) was acquired from the RCSB Protein Data Bank and chosen as the structure of the reference protein. The molecular structures of compounds were constructed YTH‐60 using Chemdraw software and saved as SDF format files.

### 2.4 Determination of thermodynamic solubility

Weigh about 1 mg of the test compound into 1.5 mL centrifuge tube and add 0.5 mL various solvents, sonicating for 5 minutes (if the compound is completely dissolved, then added again test compound until a little solid remains). Take the supernatant after centrifugation, diluting to a suitable concentration using and measuring absorbance values by UV spectrophotometer. Weigh 10 mg compound accurately of the test compound into 1.5 mL centrifuge tube and add 1.2 mL methanol solution to prepare 8.3 mg·mL^−1^ mother solution. Take the above described mother liquor to prepare the solution in concentrations of 8.3, 4.2, 2.1, 1.0, 0.52, 0.26 mg·mL^−1^ then measure absorbance value. With concentration c (mg·mL^−1^) as the abscissa, absorbance A as the ordinate, mapping and fitting line to get the standard curve (The correlation coefficient should be greater than 0.998). Calculate solubility according to the standard curve of the linear equation.

### 2.5 Kinase profile assay and IC_50_ test

The in vitro kinase enzymatic inhibition assays were carried out by the Kinase Profiling Services provided by Eurofins (UK). The detailed protocol descriptions can be provided by the website (https://www.eurofinsdiscoveryservices.com).

### 2.6 Cell lines and cell culture

The human alveolar epithelial A549 cells were purchased from American Type Culture Collection (ATCC). NIH‐3T3 (mouse embryonic fibroblast) was purchased from the China Center for Type Culture Collection (CCTCC). Human pulmonary fibroblasts (HPF) was purchased from Sciencell. Human embryonic lung cells MRC‐5 was purchased from Shanghai Zhong Qiao Xin Zhou Biotechnology Co., Ltd. The normal human liver cell line LO2 was purchased from the Cell Bank of the Chinese Academy of Science. The cells were propagated in Dulbecco's modified Eagle's medium containing 10% foetal bovine serum (v/v) and 1% antibiotics (penicillin and streptomycin) in 5% CO_2_ at 37°C.

### 2.7 Cell viability assay

The cell viability of YTH‐60 and Nintedanib treatment was performed by MTT and CCK8 assay. Briefly, the exponentially growing cells were seeded in 96‐well (2‐5 × 10^3^ cells·well^−1^) and cultured for 24 hours. The cells were treated with different concentrations of YTH‐60 (0, 0.625, 1.25, 2.5, 5, 10 µmol·L^−1^), after treatment for 24, 48 and 72 hours, respectively, a volume of 20 μL of 5 mg·mL^−1^ MTT solution or 10 μL CCK‐8 solution was added to each well and incubation for 2‐4 hours at 37°C. Finally, the 96‐well plates were read on Multiskan FC microplate spectrophotometer at 570 or 450 nm wavelength.

### 2.8 Western blot analysis

Samples of cells and mouse lung tissues were lysed in RIPA buffer with a protease and phosphatase inhibitors (Selleckchem). The protein concentrations in the cell lysate were measured by the Lowry method. Approximately 50 μg of total protein was separated by SDS‐PAGE and then transferred to polyvinylidene fluoride (PVDF) membranes (Amersham Bioscience). After the membranes incubation with the specific primary and secondary antibodies, the reactive bands were detected with enhanced chemiluminescent substrate to horseradish peroxidase.

### 2.9 Immunofluorescence assay

NIH‐3T3 and HPF cells were cultured in 24‐well plates, and treated with YTH‐60 (5 μmol·L^−1^) and TGF‐β_1_ (5 ng·mL^−1^) for 24 hours. The cells were successively washed with phosphate‐buffered saline (PBS), fixed with 4% paraformaldehyde for 15 minutes, permeabilized with 0.3% Triton X‐100 for 20 minutes at room temperature, blocked for 30 minutes with 5% BSA, and then incubated with specific primary antibodies overnight at 4°C. After a wash step, the cells were incubated with specific secondary antibodies for 1.5 hours at room temperature. Then, wash the sample with PBS, drip anti‐fluorescence quencher, and use LSM 510 laser confocal microscope to acquire images (Zeiss confocal microscope).

### 2.10 Wound healing assay

A549 cells (1 × 10^6^ well^−1^) were grown on 6‐well plates in 2 mL 10% FBS DMEM medium. Making a scratch with a sterile 200 μL microtube tip after 24 hours and the scratched cells were washed gently with serum‐free DMEM. Then, TGF‐β_1_ or YTH‐60 added to their corresponding wells and cultured for 24 hours. Each picture was imaged at three different locations. ImageJ software was used to estimate the percentage of the original width by measuring the width of the scratches.

### 2.11 Quantitative real‐time PCR (qPCR)

Total RNA of lung tissues or cells was extracted by using AxyPrep Multisource Total RNA Miniprep Kit (Axygen). Reverse transcription was carried out with *Evo M‐MLV* RT Kit with gDNA Clean (Accurate, AG11711). qRTPCR was conducted using SYBR Green *Pro Taq* HS (Accurate, AG11701). The PCR primers used were synthesized by Genewiz and listed in Table S1.

### 2.12 BLM‐induced mouse models of pulmonary fibrosis

Male C57BL/6 mice (8‐10 weeks old) were purchased from HFK Bioscience Co. Ltd. All animal experiments were permitted by the Institutional Animal Care and Treatment Committee of Sichuan University in China (New Permit Number: 20190524003). Lung fibrosis was induced using BLM treatment (2 mg·kg^−1^). All C57BL/6 mice were divided into five groups (n = 10): a BLM + Nintedanib group (Nintedanib, 30 mg·kg^−1^), a BLM + YTH‐60 group (YTH‐60, 15 mg·kg^−1^ and 30 mg·kg^−1^), a control group (Sham), and a bleomycin group (Vehicle). At the beginning, the mice were anaesthetized using 10% chloral hydrate, dissolved BLM with saline and administered intratracheally. To evaluate the preventive effect, the next day after BLM challenge, YTH‐60 and Nintedanib were administered intraperitoneally daily for 13 days (fibroproliferation) to the mice. All mice were sacrificed 24 hours after the last drug injection, the lungs and other organs were taken for the subsequent analysis. To test the therapeutic effect, after the 7th day of BLM treatment, mice were injected intraperitoneally with YTH‐60, Nintedanib and vehicle daily for 7 consecutive days (fibroproliferation).

### 2.13 Bronchoalveolar lavage fluid

After the animals were anaesthetized, rinsing the lungs with 1 mL of normal saline three times and collected bronchoalveolar lavage fluid. The total living cell count was performed by an ADVIA 2120i.

### 2.14 Histopathological staining

The left lung was detached and immediately fixed in 4% paraformaldehyde for 72 hours and then embed with paraffin wax. A microtome was used to prepare paraffin sections (3‐5 μm), after deparaffinized the tissue sections were stained with H&E and Masson's trichrome. Ashcroft scoring was used to examine the severity of pulmonary fibrosis.^20^ The image analysis‐based system to quantify collagen deposition was described previously.^21^

### 2.15 Hydroxyproline assay

The hydroxyproline measurement kit (Nanjing Jiancheng Bioengineering Institute) was used for the measurement of hydroxyproline. Collected approximately 30 mg (wet weight) of lung tissues, and added 1 mL of alkaline hydrolysate and boil the tissue for 20 minutes with continuous stirring. Adjust the pH to 6.0‐6.8 with the reagents provided. After being adsorbed on activated carbon, about 3‐4 mL of supernatant was collected for measurement. The hydrolysate was centrifuged at 900 *g* for 10 minutes. Then, carefully remove about 1 mL of supernatant for measurement based on the manufacturer's instructions.

### 2.16 Flow cytometry

We analysed the percentage of MDSCs and macrophages by Flow cytometry (FCM).^22^ Use scissors to cut approximately 30 mg of mouse lung tissues into small pieces and use 5 mL of collagenase‐IV with a concentration of 1 mg·mL^−1^, digested at 37°C for at least 1 hour. Centrifuge at 400 *g* for 5 minutes and discard the supernatant. Then, add another 5 mL PBS to resuspend, after precipitation, taken the supernatant and mixed with the corresponding fluorochromium binding antibody, incubate for 30 minutes in the dark at 4°C (MDSCs with PE^−^CD11b^−^, FITC^−^Gr1^−^ conjugated dual labelling and macrophages with PE^−^F4/80^−^, FITC^−^CD11b^−^ conjugated dual labelling). The data were acquired with a FACS Canton II NovoCyte (ACEA).

### 2.17 Enzyme‐linked immunosorbent assay

IL‐2, IL‐4, IL‐10, IL‐17A, TNF‐α and IFN‐γ levels in mouse serum were determined using a mouse Th1/Th2/Th17 Cytokine Kit (BD Biosciences) according to the manufacturer's instructions. In brief, preparing cytokine standards, cytokine capture microsphere suspensions and serum samples, 50 μL of capture microsphere suspension, 50 μL of mouse TH1/TH2/TH17 PE signal antibody, 50 μL of the corresponding mouse TH1/TH2/TH17 cytokine dilution and 50 μL of test samples were added in each tube then incubated at room temperature in the dark 3 hours. Wash and centrifuge at 200 *g* for 5 minutes, discard the supernatant and add 300 μL of washing solution to each tube, resuspend the microspheres, analyse the samples using FCM, and analyse the calculation results using BDCBA software.

### 2.18 Pharmacokinetics study

The appropriate amount of YTH‐60 was accurately weighed, added to the final volume of 5% DMSO, 40% PEG400 and 55% normal saline, and vortexed or ultrasonicated to fully mix. Then, 0.4 and 2 mg·mL^−1^ of clarified dosing solution was used for intravenous administration and oral gavage administration. SD rats were transferred from the Experimental Animal Library (999 M‐017), Shanghai Xipuer‐Beikai Experimental Animal Co. Ltd. After oral, intravenous or intragastric administration, blood was collected by jugular vein or other suitable methods. Approximately 0.20 mL of each sample was collected, in the presence of heparin sodium as an anticoagulant. After the blood samples were collected, they were placed on ice and centrifuged to separate plasma within 2 hours (centrifugation conditions: centrifugal force 6800 *g*, 6 minutes, 2‐8°C). The collected plasma samples were stored at −80°C before analysis by LC‐MS/MS‐18. After analysis, the remaining plasma samples were stored at −80°C for a period of 1 month.

### 2.19 Statistical analysis

All in vitro studies were performed at least three times. Results are expressed as mean ± SD and *P*‐values were determined by two‐tailed Student's *t* test for comparison of two groups. Statistically significant *P* values were considered at: *P < .05, **P < .01 and ***P < .001; ^#^ *P* < .05, ^##^<0.01 and ^###^ *P* < .001.

## 3 RESULTS

### 3.1 Design, synthesis, screening, kinase inhibition profile and molecular modelling studies of YTH‐60

A total of 550 novel multi‐kinase compounds were designed through computer aided drug design (CADD). There are 55 lead compounds ranked in the top 10% according to Ludi Energy Estimates, and preliminary screening has been carried out through the kinase inhibition test (date not shown). Among these, (*E*)‐*N*‐(4‐(2‐(6‐(2,6‐dichloro‐3,5‐dimethoxyphenyl)‐1*H*‐indazol‐3‐yl)vinyl)phenyl)‐*N*‐methyl‐2‐(4‐methylpiperazin‐1‐yl)acetamide (YTH‐60) displays robust kinase and cellular inhibitory activity in vitro. The structural formula and synthesis route of YTH‐60 are shown in Figure 1A and Figure S1. To clarify the inhibitory effects of YTH‐60 on multiple proteins, an in vitro kinase assay was performed (Table 1). YTH‐60 robustly inhibited FGFR1‐3, with IC_50_ values of 4‐7 nmol·L^−1^. In addition, YTH‐60 inhibited VEGFR1‐3 and had an IC_50_ of 61‐123 nmol·L^−1^ and exhibited relatively weak kinase inhibition of PDGFRα and PDGFRβ. In order to further understand the activity of YTH‐60 against FGFR1 kinase, we performed molecular docking into FGFR1 kinase. Through computer simulation and molecular docking methods, the interaction mode between YTH‐60 and FGFR1 kinase domain (PDB ID: 5UR1) was obtained (Figure 1B,C). It is observed that YTH‐60 forms strong hydrogen bond interactions with surrounding amino acid residues such as GLU531, GLU562, LYS566, GLY567 and ASP641. YTH‐60 forms hydrophobic interactions with the surrounding amino acid residues such as LEU484, VAL492, ALA512, LYS514, ILE545, VAL561, ALA564, LEU630 and ALA640. LYS514 of YTH‐60 forms an electrostatic force. The above results speculate that YTH‐60 could bind with protein stably. These results shown that YTH‐60 was a potent multi‐target inhibitor especially targeting FGFR1. In addition, YTH‐60 has better solubility than Nintedanib (Table 2). Therefore, we chose Nintedanib as a positive control to evaluate the efficacy of YTH‐60.

**TABLE 1:** In vitro profile of YTH‐60 against a panel of 11 kinases

Kinase	IC_50_ ^a^ (nmol·L^−1^)
Abl (h)	27
FGFR1 (h)	4
FGFR2 (h)	7
FGFR3 (h)	9
FGFR4 (h)	424
Flt1 (h)/VEGFR1	123
Flt3 (h)	297
Flt4 (h)/VEGFR3	64
KDR (h)/VEGFR2	61
PDGFRα (h)	921
PDGFRβ (h)	>3000
Src (1‐530) (h)	633

^a^ The biochemical tests were provided by Eurofins Discovery. All data were obtained by double testing.

**TABLE 2:** Compound solubility

	Solubility^a^ (mg·mL^−1^)					
	Water	Normal saline	Ethyl alcohol	Acetone	Ethyl acetate	Acetonitrile
Nintedanib	0.28 ± 0.015	1.93 ± 0.18	12.48 ± 2.20	9.83 ± 0.42	9.75 ± 0.35	3.525 ± 0.064
YTH‐60	1.71 ± 0.14	12.71 ± 0.15	77.02 ± 1.53	83.10 ± 0.89	101.20 ± 4.02	18.31 ± 1.20

^a^ All data were obtained by double testing.

### 3.2 YTH‐60 inhibits fibroblast activation and proliferation

**FIGURE 2:**
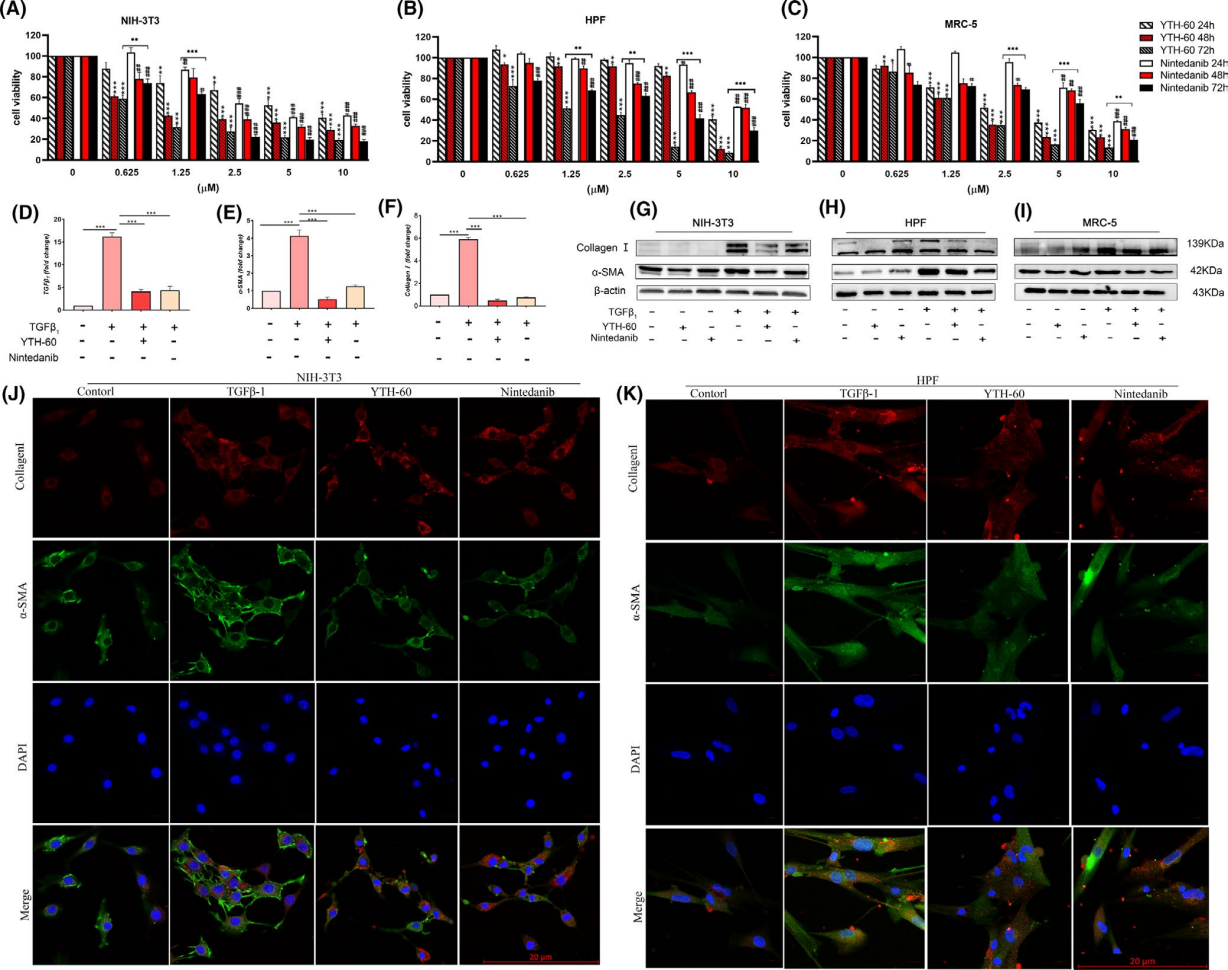
YTH‐60 inhibits fibroblast activation and proliferation. A‐C, NIH‐3T3, HPF and MRC‐5 were treated with different concentrations of YTH‐60 and Nintedanib for 24, 48 or 72 h and cell viability was measured by the CCK8 assay. The values are expressed as the mean ± SD (n = 3); *P < .05; **P < .01; *** *P* < .001 compared with YTH‐60 control; ^#^ *P* < .05; ^##^ *P* < .01; ^###^ *P* < 0.001 compared with Nintedanib control. D‐F, Real‐time qPCR analysis of collagen Ⅰ, α‐SMA and TGF‐β1 in NIH‐3T3 cells treated with 5 ng·mL^−1^ TGF‐β1 and 2.5 μmol·L^−1^ YTH‐60 or Nintedanib for 24 h. The values are expressed as the mean ± SD, n = 3; *P < .05; **P < .01; ***P < .001. G‐I, NIH‐3T3, HPF and MRC‐5 were stimulated with TGF‐β1 by 2.5 and 5 μmol·L^−1^ YTH‐60 administration for 24 h, and expression of α‐SMA and collagen Ⅰ using western blot. J‐K, expression of α‐SMA and collagen Ⅰ was visualized by immunofluorescence, Scale bar = 50 μm

To further confirm the effects of YTH‐60, we studied the time‐ and concentration‐dependent effects of YTH‐60 on the proliferation of mouse embryonic fibroblast cell lines (NIH‐3T3), human pulmonary fibroblast cell lines (HPF) and human embryo lung fibroblast (MRC‐5). After these cell lines were exposed to YTH‐60 or Nintedanib for 24‐72 hours, their cell viability decreased with the increase of drug concentration and exposure time. These results demonstrate that YTH‐60 inhibits cell proliferation in a time‐ and concentration‐dependent manner. These three fibroblast lines were much more sensitive to YTH‐60 than Nintedanib (Figure 2A‐C). The cytotoxicity of YTH‐60 in vitro was confirmed using the LO_2_ cells. YTH‐60 inhibited the viability of LO2 cells less than Nintedanib after 24 hours exposure (Figure S2A,B). Real‐time qPCR and western blot analysis further demonstrated the inhibitory effects of YTH‐60 on fibroblast activation. NIH‐3T3 cells were treated with TGF‐β_1_ and 2.5 μmol·L^−1^ YTH‐60 for 24 hours, and co‐culture with TGF‐β_1_‐treated NIH‐3T3 cells increased the mRNA levels of α‐SMA, collagen I and TGF‐β_1_, while YTH‐60 reduced this effect (Figure 2D‐F). Furthermore, YTH‐60 attenuated the expression of fibrosis‐associated α‐SMA and collagen I induced by TGF‐β_1_ stimulation (Figure 2G‐I). Likewise, an immunofluorescence assay was conducted to evaluate the anti‐fibrotic effects of YTH‐60 in NIH‐3T3 and HPF cells. After treatment with YTH‐60 for 24 hours the expression of α‐SMA and collagen I was lessened (Figure 2J,K). These results indicated that YTH‐60 could inhibit fibroblast activation and proliferation.

### 3.3 YTH‐60 exhibited anti‐fibrotic activity by inhibiting FGFR and TGF‐β/Smad pathways

**FIGURE 3:**
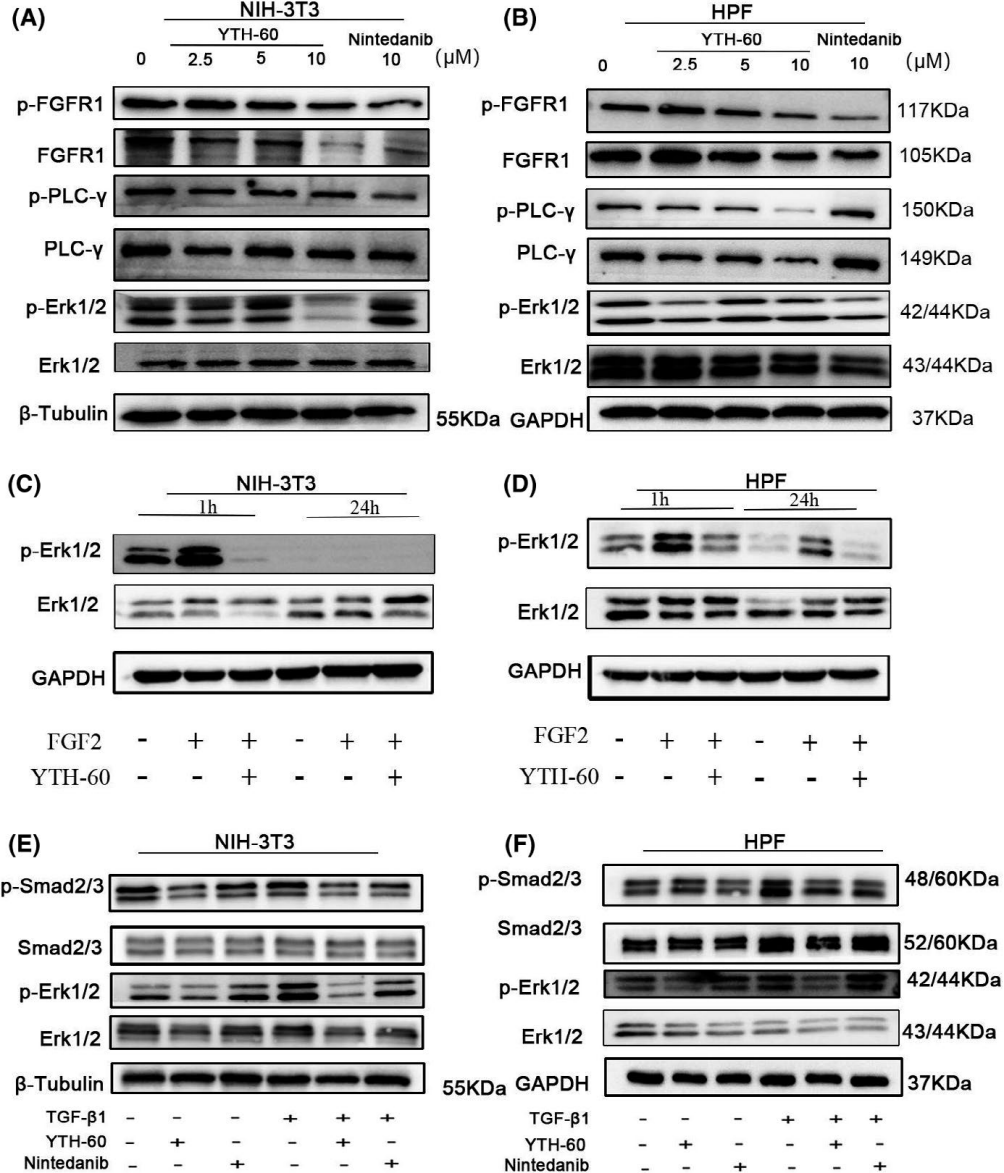
YTH‐60 exhibited anti‐fibrotic activity by inhibiting FGFR and TGF‐β/Smad pathways. NIH‐3T3 (A) and HPF cells (B) were stimulated with TGF‐β1 (5 ng·mL^−1^) and YTH‐60 (2.5, 5, 10 μmol·L^−1^) or Nintedanib for 24 h and subjected to western blot analysis. C, D, NIH‐3T3 and HPF cells were stimulated with FGF2 (50 ng·mL^−1^) and YTH‐60 (5 μmol·L^−1^) for 1 and 24 h and subjected to western blot analysis. E, F, Effect of YTH‐60 or Nintedanib (5 μmol·L^−1^) on TGF‐β1‐induced phosphorylation protein expression of Smad2/3 and ERK1/2 in NIH3T3 and HPF cells. GAPDH and β‐Tubulin were used as a loading control

In order to further evaluate the cellular activity of YTH‐60 targeting FGFR kinase, we analysed its effects on the phosphorylation of FGFR, PLCγ and Erk. As shown in Figure 3A,B, 10 μmol·L^−1^ YTH‐60 inhibited the phosphorylation of FGFR1 in fibroblasts. Like the inhibition of FGFR1 phosphorylation, the phosphorylation of PLCγ and Erk was also inhibited. Although the expression of non‐phosphorylated FGFR1, PLCγ and Erk1/2 is also reduced, the effect on phosphorylation is more obvious. In order to further verify the FGFR pathway, we stimulated fibroblasts with FGF2 for 1 and 24 hours in vitro. As shown in Figure 3C,D, the expression of downstream protein p‐ERK1/2 increased after activation of the FGFR pathway, while YTH‐60 could reduce its expression. These results indicate that YTH‐60 shows effective blocking of FGFRs signalling. In order to further study the target molecule of YTH‐60 in the signal pathway mediated by TGF‐β_1_, we found the phosphorylation of Smad‐2/3 and Erk1/2. Fibroblasts were incubated with or without TGF‐β_1_ in the presence or absence of YTH‐60 for 24 hours. TGF‐β_1_ stimulation significantly enhances the phosphorylation levels of Smad2/3, whereas treatment with YTH‐60 significantly reduced this phosphorylation (Figure 3E,F). Meanwhile, YTH‐60 inhibited the expression of phosphorylated Erk1/2. Therefore, it could be inferred that YTH‐60 ameliorated fibrosis by inhibiting both FGFR and TGFβ/Smad‐dependent pathways.

### 3.4 YTH‐60 blocks TGF‐β_1_‐induced EMT

**FIGURE 4:**
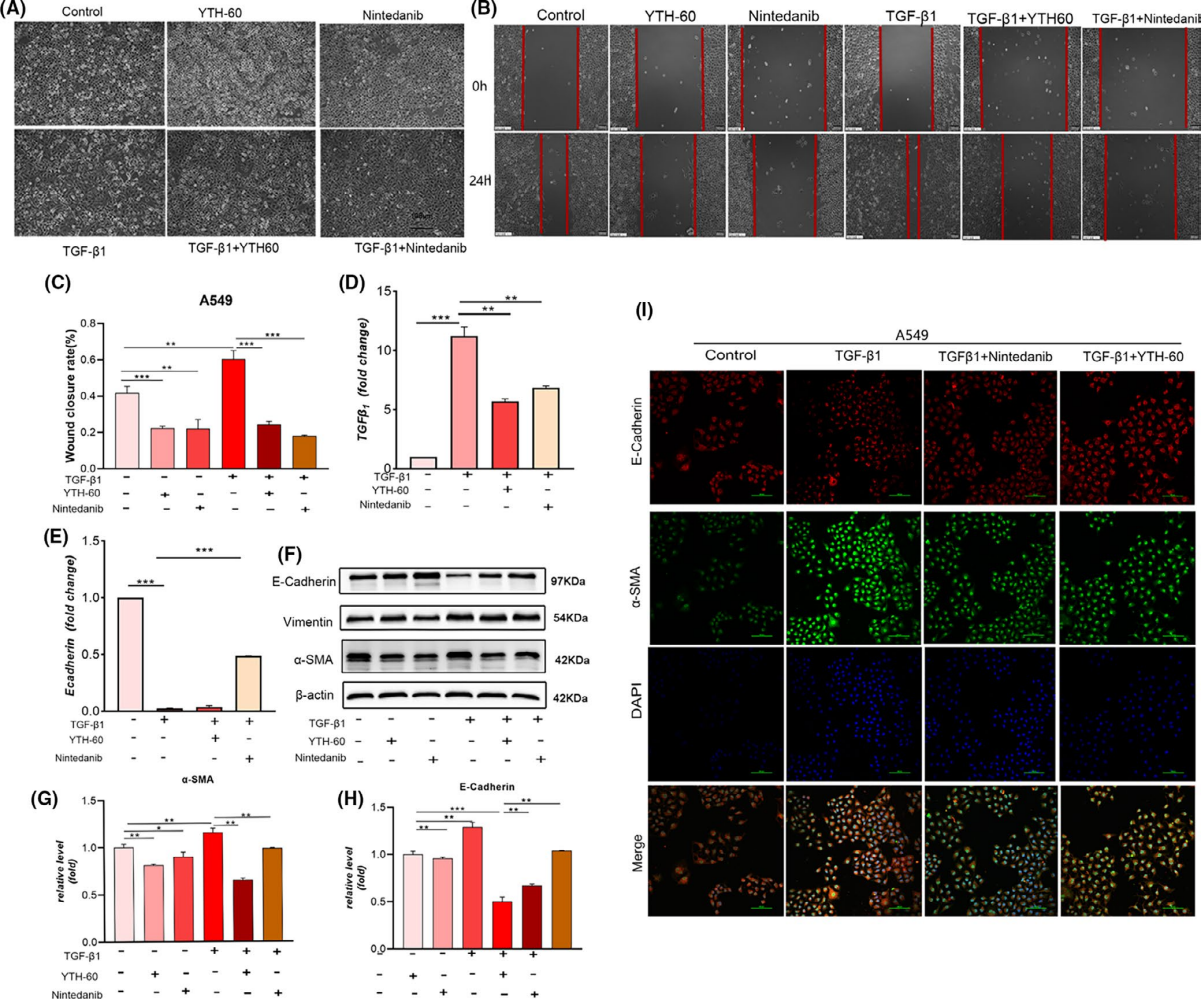
YTH‐60 blocks TGF‐β1 induced EMT in A549 cells. A, The morphological changes were imaged using phase contrast microscopy. B, A549 cells were seeded in a 6‐well plate for 24 h, then starved with serum‐free medium for 6 h, and the medium was replaced with 5 ng·mL^−1^ TGF‐β1 fresh medium. A scratch was made using a sterile 200 μL micropipette tip. One hour later, 5 μmol·L^−1^ YTH‐60 was added. After 24 h, the cells were photographed by a microscope (Scale bar = 100 μm). C, Healing rate (%) = (wound area at 24 h/wound area at 0 h) × 100% (Scale bar = 100 μm). Data are presented as mean ± SD, n = 3, *P < .05; **P < .01 and ***P < .001, Scale bar = 100 μm. D, E, Quantitative real‐time qPCR analysis was used to measure the mRNA levels of E‐cadherin and TGF‐β1 in A549 cells stimulated with TGF‐β1 followed by 5 μmol·L^−1^ YTH‐60 administration for 24 h. F, Expression changes of α‐SMA, E‐cadherin and vimentin using western blot in A549 cells. G, H, Analysed by densitometry. Data are presented as mean ± SD, n = 3, *P < .05; **P < 0.01 and ***P < .001. I, The levels of E‐cadherin and α‐SMA were evaluated by immunofluorescence experiment. Scale bar = 100 μm

Next, we want to further explore whether YTH‐60 could inhibit the EMT. First, we noticed that YTH‐60 suppressed the viability of A549 cells (Figure S2C,D). Subsequently, the morphological changes of A549 cells induced by TGF‐β1 were observed from ovoid epithelial cells to spindle‐shaped fibroblasts cells (Figure  4A). Compared with the control A549 cells, the cell migration activity was enhanced after TGF‐β_1_ treatment. The combined treatment of YTH‐60 and TGF‐β_1_ significantly inhibited cell migration than TGF‐β_1_ treatment alone, suggesting that YTH‐60 could inhibit cell migration (Figure 4B,C). Further study showed that co‐culture with TGF‐β_1_‐treated A549 cells decreased the mRNA levels of E‐cadherin and increased TGF‐β_1_, while YTH‐60 reversed this effect (Figure 4D,E). In A549 cells treated with TGF‐β_1_, YTH‐60 raised the expression of epithelial marker E‐cadherin and inhibited the expression of mesenchymal markers α‐SMA and vimentin (Figure 4F‐H). The immunofluorescence results further showed that compared with TGF‐β_1_ treatment alone, YTH‐60 treatment could increase the expression of E‐cadherin and suppress the expression of α‐SMA (Figure 4I).

### 3.5 Pharmacokinetic study of YTH‐60 after IV and PO administration to SD rats

**FIGURE 5:**
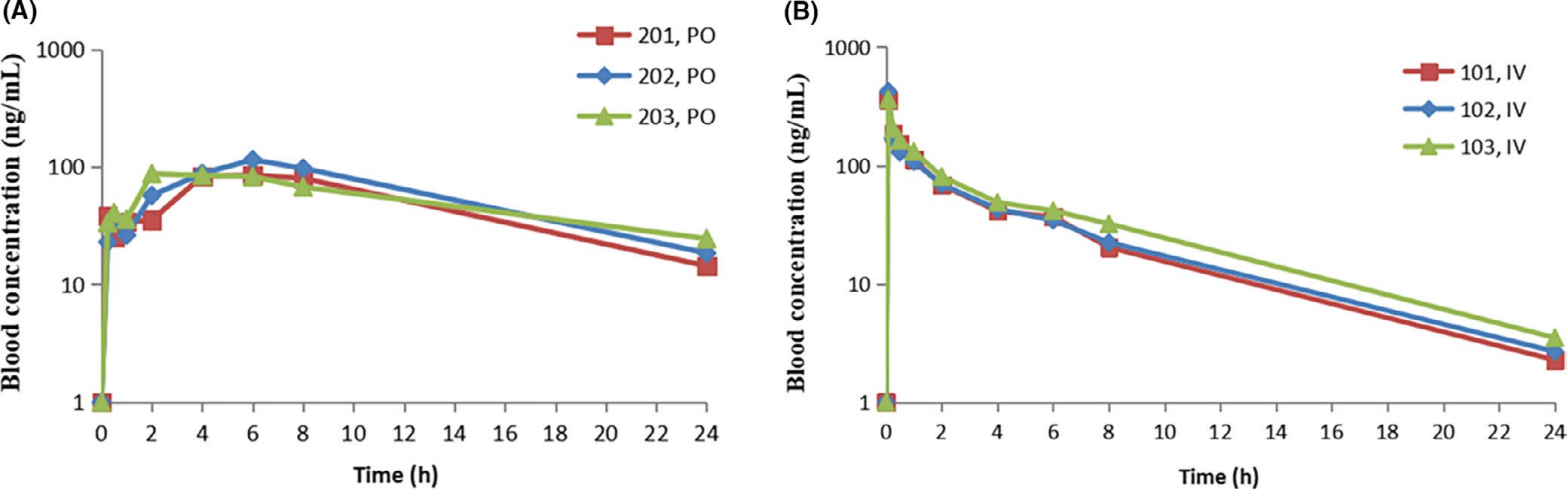
Blood concentration‐time curve of the YTH‐60 after oral administration and blood concentration‐time curve of the YTH‐60 after intravenous infusion

To evaluate the efficacy of YTH‐60 in animal models, we first examined the pharmacokinetic profile of YTH‐60. Blood concentration‐time curve of the compound after oral administration and intravenous infusion are shown in Figure 5A,B. After an IV dose, the mean half‐life was 4.92 hours, and the mean AUC(_0‐t_) was 772.62 h·ng·mL^−1^. The mean bioavailability was 17.86% for 20 mg·kg^−1^ YTH‐60 by oral administration. The mean half‐life was 8.03 hours, and the mean AUC(_0‐t_) was 1379.73 h·ng·mL^−1^ (Table 3 and Table S2). While, the mean bioavailability was 11.9% for 50 mg·kg^−1^ Nintedanib by oral administration.^23^ This result suggests that YTH‐60 might be a potential drug candidate with low toxicity and has better absorption than Nintedanib.

**TABLE 3:** In vivo pharmacokinetic parameters of YTH‐60

Parameter	YTH‐60	
	*i.v*. (2 mg·kg^−1^)	*p.o*. (20 mg·kg^−1^)
AUC_0‐t_ (ng·m^−1^·h)	772.62 ± 99.37	1379.73 ± 152.10
AUC_0‐∞_ (ng·mL^−1^·h)	792.72 ± 104.20	1611.00 ± 177.20
MRT_0‐t_ (h)	4.50 ± 0.28	8.38 ± 0.31
MRT_0‐∞_ (h)	5.16 ± 0.36	12.36 ± 2.67
*C*_max_ (ng·mL^−1^)	380.42 ± 37.00	95.85 ± 16.56
*T*_max_ (h)	0.08 ± 0	4.670 ± 2.31
*T*_1/2_ (h)	4.920 ± 0.17	8.030 ± 2.16
*F* (%)	‐	17.86 ± 1.97
Cl	2550.42 ± 313.43	‐

Note

‐: Not applicable.

### 3.6 YTH‐60 prevents BLM‐induced pulmonary fibrosis

**FIGURE 6:**
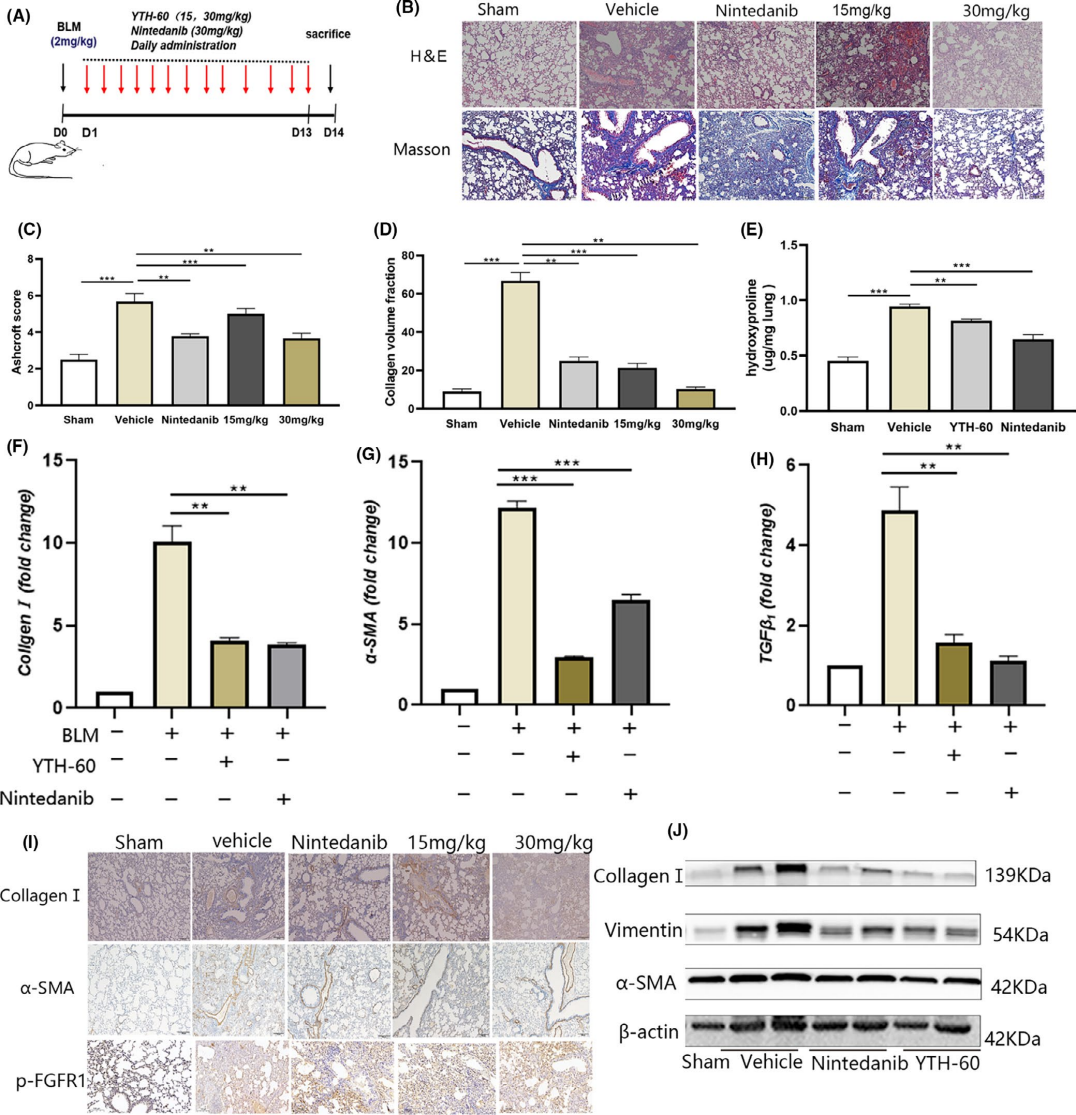
YTH‐60 alleviates BLM‐induced pulmonary fibrosis in mice. A, The experimental schedule of YTH‐60 treatment for BLM‐induced pulmonary fibrosis. B, Representative images of YTH‐60 ameliorated the histopathological changes. Pulmonary tissues stained with H&E or Masson staining were shown from sham, BLM, BLM + YTH‐60 (15, 30 mg·kg^−1^) and BLM + Nintedanib (30 mg·kg^−1^) group. C, YTH‐60 treatment reduced the pulmonary fibrosis Ashcroft scoring. D, Morphometric analysis of collagen was used to measure the degree of lung fibrosis. E, Hydroxyproline content of lung tissues. Data are presented as mean ± SD, n = 3‐8 for each group, *P < .05; **P < .01; ***P < .001. F‐H, The mRNA levels of Collagen Ⅰ,α‐SMA and TGF‐β1 in the lung tissues was measured by Quantitative real‐time qPCR. Data are expressed as the mean ± SEM (n = 3 mice per group). I, Immunohistochemical determination of Collagen Ⅰ, α‐SMA and p‐FGFR expression in the lung tissues. J, The collagen Ⅰ, α‐SMA and vimentin of lung tissues were performed by western blotting

To investigate the anti‐fibrosis effect of YTH‐60 in vivo, mice were administered intratracheally with BLM (2 mg·kg^−1^) and treated with different doses of YTH‐60 (15 and 30 mg·kg^−1^) and the Nintedanib as positive control (30 mg·kg^−1^) (Figure 6A). BLM enhanced pulmonary inflammatory responses and destroyed lung architecture when compared to the control group, mice with collapsed alveolar spaces and thickened alveolar septa after BLM treatment, as evidenced by semiquantitative analysis using fibrotic Ashcroft histological evaluation of H&E staining (Figure 6B,C). In addition, mice treated with BLM illustrated notable deposition of collagen, as indicated by Masson‐staining and measuring hydroxyproline (Figure 6B,D,E). Importantly, these features of BLM‐induced inflammation and fibrosis were reduced after YTH‐60 administration (Figure 6B‐E). Moreover, there were no obvious lesions in the heart, liver, spleen and kidney of mice in each group (Figure S3).

These data initially suggest that YTH‐60 has a certain protective effect on pulmonary fibrosis. Next, we further investigated the anti‐fibrotic effect of YTH‐60. We detected the levels of endogenous α‐SMA, collagen I and TGF‐β_1_ mRNA in the lung tissues of BLM‐treated mice. YTH‐60 could reduce the expression of these indicators (Figure 6F‐H). Immunohistochemistry also showed that YTH‐60 significantly decreased the expression of α‐SMA, collagen I and p‐FGFR1 (Figure 6I). In addition, western blot analysis further confirmed these results (Figure 6J).

### 3.7 YTH‐60 has anti‐inflammatory effects in BLM‐induced lung fibrosis

**FIGURE 7:**
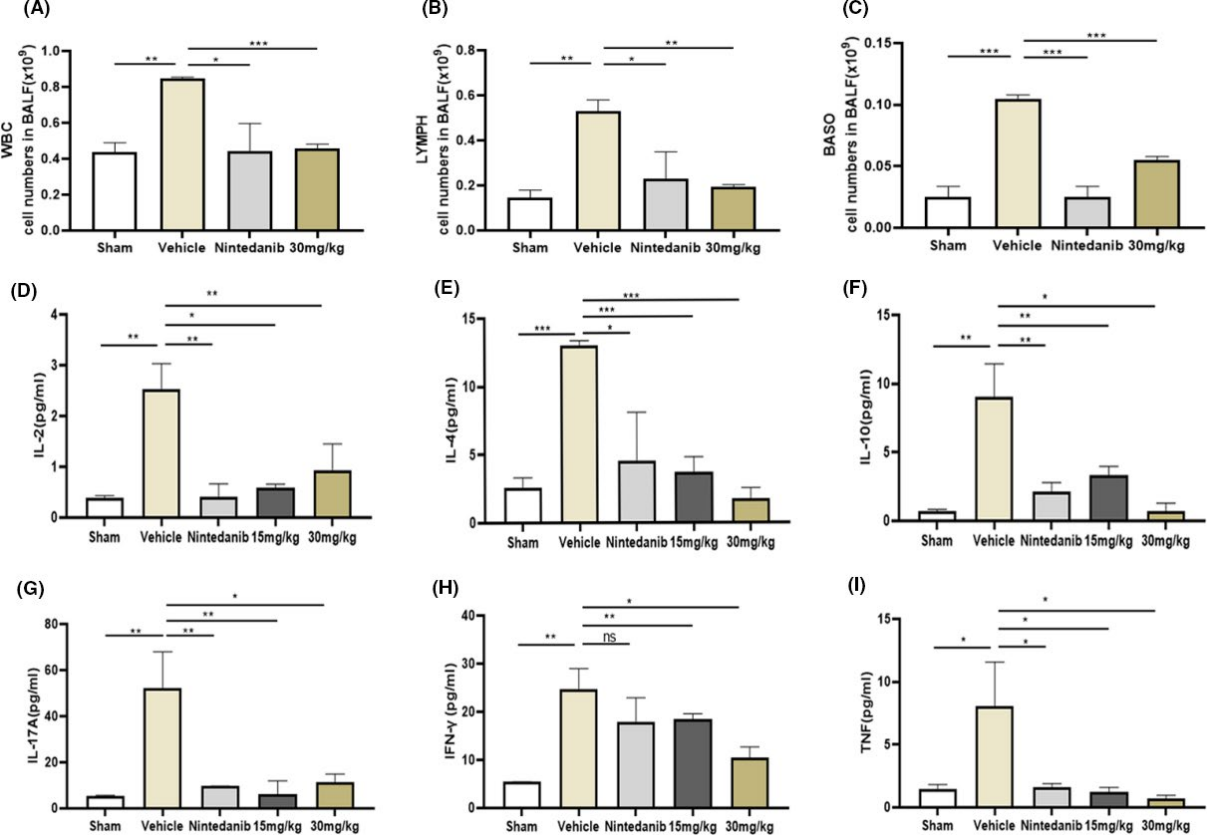
YTH‐60 possesses an anti‐inflammatory effects on BLM‐induced pulmonary fibrosis. A‐C, Bronchoalveolar lavage fluid (BALF) analysis was performed by counting the number of various cells. D‐I, The cytokines in the serum of mice were analysed by ELISA. Data are expressed as mean ± SD, n = 3‐8, *P < .05; **P < .01; ***P < .001, NS indicates no significant differences were found among groups

Because BLM could cause inflammatory cells to enter the lungs in the early stages of inflammation,^24^ we analysed the bronchoalveolar lavage fluid (BALF) of BLM mice on the 14th day after treatment. Increased infiltration of white blood cells (WBCs), Basophils (BASO) and lymphocyte (LYMPH) in the BLM mice (Figure 7A‐C) was consistent with previous research.^25^ YTH‐60 at a dose of 30 mg·kg^−1^ could significantly reduce the number of infiltrating inflammatory cells in the BALF of BLM‐induced mice. The interaction between cytokines and fibroblasts in the process of pulmonary fibrosis has become the focus of more and more research interests.^26^ Therefore, we tried to detect the expression of multiple inflammatory factors, such as TNF‐α, IFN‐γ, IL‐2, IL‐4, IL‐10 and IL‐17A. Marked increases in the production of these inflammatory cytokines were observed on day 14 after BLM exposure compared with those of sham group. However, YTH‐60 reduced the expression level of these inflammatory cytokines in the serum of BLM‐treated mice (Figure 7D‐I).

### 3.8 YTH‐60 inhibits BLM‐induced imbalance in the lung immune microenvironment

**FIGURE 8:**
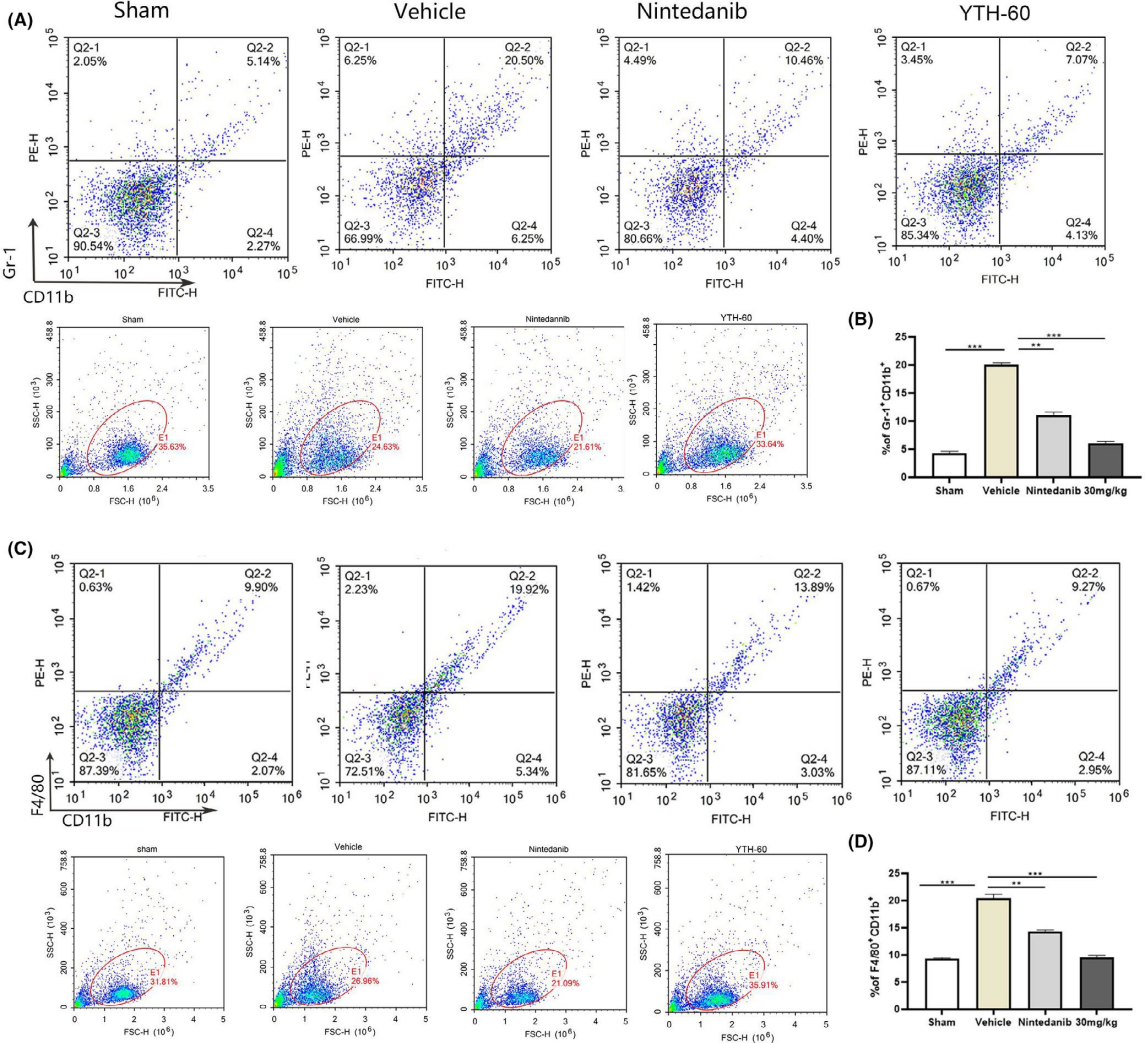
YTH‐60 inhibits bleomycin‐induced imbalance of lung immune microenvironment. A, B, Single‐cell suspensions prepared from each group lungs were analysed by flow cytometry quantified CD11b^+^Gr1^+^. C, D, YTH‐60 significantly reduced macrophages in the lung tissues from each group. Flow cytometry analysis quantified CD11b^+^F4/80^+^. Bars show mean ± SEM; three mice every group, *P < .05, **P < .01 and ***P < .001

The cells of the immune system show abundant and multifaceted contributions to IPF, including monocytes, macrophages, MDSCs and innate lymphoid cells.^27^ In this study, we treated mice with BLM for 14 days, collected lung tissue, and detected the number of MDSCs (Gr1^+^CD11b^+^) and macrophages (F4/80^+^CD11b^+^) by FCM. The results indicated that the influx of lung Gr1^+^CD11b^+^ MDSCs (Figure 8A) and CD11b^+^F4/80^+^ macrophages (Figure 8B) were markedly increased in the BLM‐treat group while decreased in the YTH‐60‐treat group. As shown in Figure 8, an average of 20.5% CD11b^+^Gr1^+^ cells and 19.9% F4/80^+^CD11b^+^ cells in the BLM‐treat group vs 5.14% and 9.9%, respectively, in the sham group. The YTH‐60 group had apparently decreased percentage of CD11b^+^Gr1^+^ and CD11b^+^F4/80^+^ cells, at a dose of 30 mg·kg^−1^, with an average of 7.07% and 9.27% in the YTH‐60 group, with an average of 10.46% and 13.89% in the Nintedanib group (30 mg·kg^−1^) (Figure 8A‐D).

### 3.9 YTH‐60 exerts anti‐fibrotic effect in BLM‐induced lung fibrosis

**FIGURE 9:**
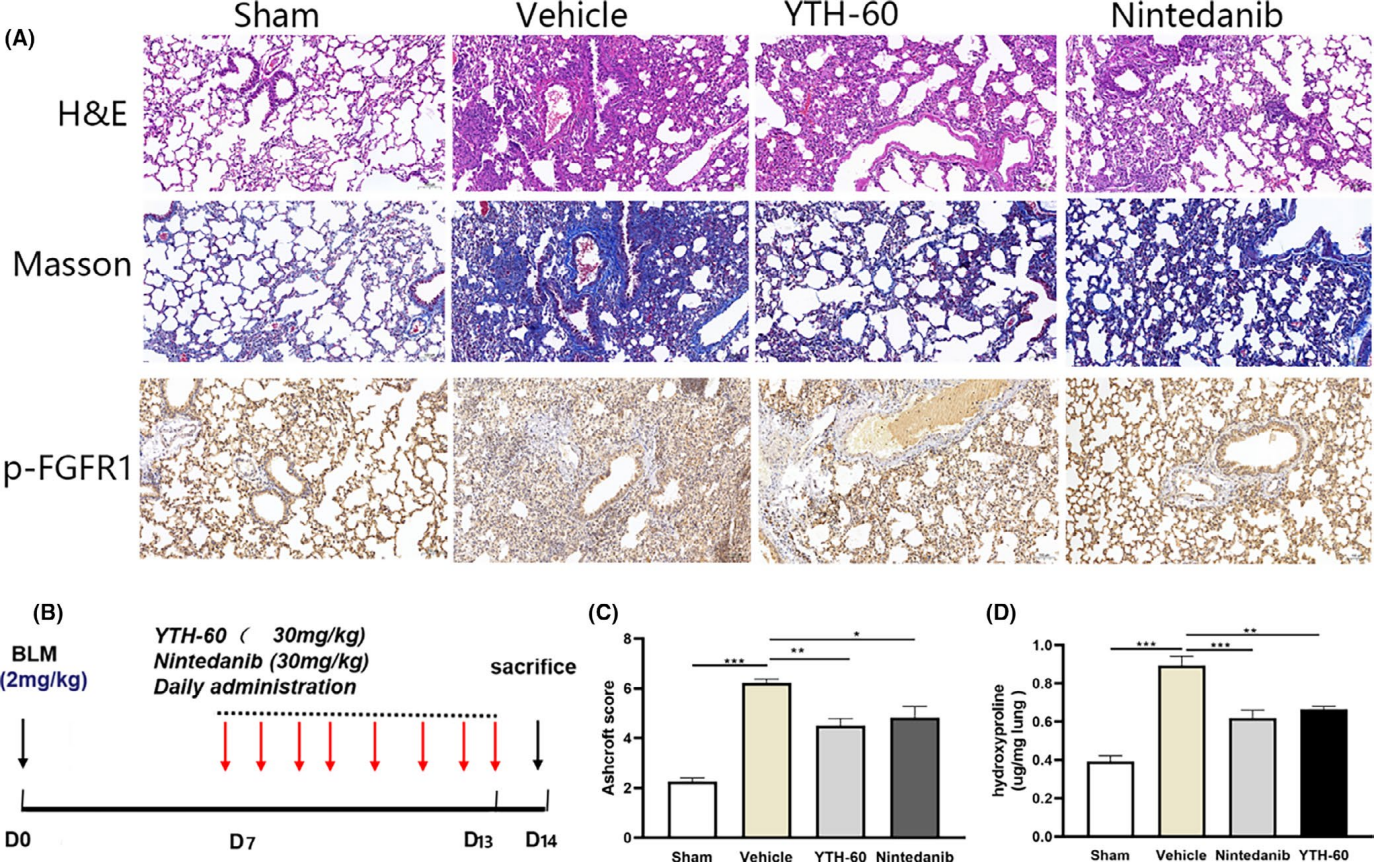
YTH‐60 exerts therapeutic role in the period of BLM‐induced pulmonary fibrosis during the fibrogenic phase. Mice were administered with BLM (2 mg·kg^−1^) and then treated once a day by YTH‐60 or Nintedanib (30 mg·kg^−1^) from day 7 to day 14 after BLM challenge. A, Lung tissues stained with H&E or Masson staining. B, The experimental schedule. C, Ashcroft scoring. D, Hydroxyproline content of lung tissues. n = 3‐8 for each group, *P < .05; ***P < .001, Data are presented as mean ± SD

It is not enough to administer the drug before the 7th day of the acute bleomycin model and only test the anti‐fibrotic compounds, so many researchers have recommend evaluating the anti‐fibrotic characteristic of any new target drugs pre‐clinical investigational drugs, especially when inflammation subsides.^28^ Here, we injected bleomycin (2 mg·mL^−1^) into mice, and then injected YTH‐60 intraperitoneally from 7th to 14th day (Figure 9A,B) after BLM instillation. Between the vehicle group and the YTH‐60 group, the administration of YTH‐60 on the 7th day of treatment caused significant differences in the fibrosis Ashcroft score (Figure 9A‐D). Therefore, the data show that YTH‐60 can not only prevent pulmonary fibrosis in the early stage of inflammation, but also has a certain therapeutic effect in the period of fibrosis.

## 4 DISCUSSION

Pulmonary fibroblasts is a complex lung disease, and the few treatment options reflect our limited understanding of the pathophysiology of pulmonary fibrosis. The differentiation of pulmonary fibroblasts into myofibroblasts is the main feature of the pulmonary fibrosis process. The activated myofibroblasts express α‐SMA stress fibres, deposit collagen, migrate and invade fibrosis foci, and become the key effector cells. Here, we demonstrated that YTH‐60 inhibited TGF‐β_1_‐mediated fibroblast differentiation in vitro. YTH‐60 inhibited the viability of a variety of fibroblast cell lines in a concentration‐dependent and time‐dependent manner, especially in NIH‐3T3 cells. YTH‐60 could efficaciously suppressed the expression of molecules related to fibrosis upregulated by TGF‐β_1_. Furthermore, YTH‐60 can simultaneously prevent and treat pulmonary fibrosis and reduce lung inflammation in mouse lung fibrosis models induced by BLM.

Earlier kinase activity assays showed that YTH‐60 has the best inhibitory effect on FGFR1, thus we choose FGFR1 as the target of drug action and used the clinical drug Nintedanib as a positive control and evaluated the effects of YTH‐60 against pulmonary fibrosis in in vivo studies. YTH‐60 has an improved inhibitory effect on a range of fibroblast cell lines and YTH‐60 had better solubility than Nintedanib. YTH‐60 exhibited an effective blockage of FGFRs signalling. This is in line with several studies that FGFR inhibitors interfere the fibrosis process induced by TGF‐β_1_,^29^ non‐specific inhibition of FGFR can reduce BLM‐induced IPF in rodents^30^ and specific FGFR1 inhibitors (NP603) can reduce rat liver fibrosis caused by tetrachlorocarbon.^31^ Studies have shown that TGF‐β_1_ mediates the differentiation of pulmonary fibroblasts into myofibroblasts through Smad‐dependent or Smad‐independent pathways. Here, we clearly demonstrated that YTH‐60 prevented lung fibrosis by modulating TGF‐β_1_/Smad pathway in vitro, evidencing by decreasing Erk1/2 and Smad2/3 phosphorylation.

It is generally believed that EMT is caused by the imbalance between lung epithelial cells and lung fibroblasts, damage to epithelial cell activation of innate immunity also can induce the differentiation of myofibroblasts.^32^ The transformation of epithelial cells into mesenchymal cells is increased,^33^ the mesenchymal marker α‐SMA was upregulated and the epithelial marker E‐cadherin was downregulated. YTH‐60 induced the upregulation of epithelial markers and reversed EMT phenotypic changes and inhibited cell migration from TGF‐β_1_ treated epithelial cells, indicating that YTH‐60 may be involved in targeting the EMT process in pathogenesis. However, many histological studies have shown that the role of the EMT in pulmonary fibrosis is inconsistent, largely due to the poverty of effective methods to test the dynamic and transient states of EMT due to the lack of clear markers to assess the interstitial phenotype.^34^, ^35^

Bleomycin can induce lung damage, and subsequent fibrotic changes that similar to pathological characteristics of human pulmonary fibrosis.^28^, ^36^ On day 14 after BLM injury, H&E and Masson staining demonstrated that the BLM‐induced pulmonary fibrosis model was successfully prepared and YTH‐60 at 30 mg·kg^−1^ has a good preventive effect on BLM‐induced pulmonary fibrosis in mice, while the 15 mg·kg^−1^ dose seems to be less effective. Histological analysis confirmed the anti‐inflammatory effect of YTH‐60 which lessened alveolar thickness, reserved alveolar integrity, and diminished collagen accumulation. More importantly, mice received YTH‐60 treatment every day for 14 consecutive days displayed pro‐fibrosis markers were significantly reduced.

Despite the controversies, there is increasing evidence that there is a broader link between inflammation and pulmonary fibrosis. The pro‐inflammatory cytokines, including IL‐2, IL‐4, IL‐10, IL‐17A, and TNF‐γ, released by recruited inflammatory cells, which can also be regarded as a pro‐fibrotic factor to induce fibrosis. In the present study showed that YTH‐60 could significantly reduce the number of WBC, eosinophils and lymphocytes in BALF. Next, we found that daily intraperitoneal injection of YTH‐60 from the second day after BLM treatment could effectively prevent BLM‐induced cytokine secretion. In order to distinguish it from YTH‐60 anti‐inflammatory, we injected YTH‐60 intraperitoneally on the 7th day of establishing the bleomycin mouse model. YTH‐60 could also slow down pulmonary fibrosis during the period when lung inflammation subsides. The results of H&E and Masson staining demonstrated that YTH‐60 (30 mg·kg^−1^) has a good therapeutic effect on BLM‐induced mouse lung fibrosis.

In the field of tissue damage and inflammation, MDSCs and macrophages are essential for controlling the immune response. Alveolar macrophages are a significant source of TGF‐β_1_.^32^ There is evidence suggesting that MDSCs accumulation is a result of chronic inflammation and the cytokines produced as a result of this inflammation.^33^ Our findings also suggested that YTH‐60 significantly reduced the number of macrophages and MDSCs in mouse lung tissue.

Our findings also suggested that YTH‐60 attenuates collagen accumulation in lung tissue, which may be a follow‐up result of inhibiting lung inflammation and lung injury. This may partly explain why inflammation in the lungs is reduced. Therefore, inhibiting leukocyte recruitment directly affects inflammation and tissue repair, which may partially explain the preventive effects of YTH‐60 on BLM‐induced pulmonary fibrosis. Therefore, we proved that YTH‐60 effectively improves pulmonary fibrosis, in part because YTH‐60 reduces the expression and activation of some inflammatory cytokines and fibrotic cytokines, thereby reducing multiple interactions between them. We also demonstrated that the anti‐fibrotic effects of YTH‐60 may be due to several mechanisms, including EMT, FGFR and TGF‐β/Smad signalling pathway. In summary, in two models of pulmonary fibrosis, our findings suggest a potential treatment for pulmonary fibrosis and this compound is expected to provide a clinical strategy for idiopathic pulmonary fibrosis in the future.

## CONFLICT OF INTEREST

The authors declare that they have no known competing financial interests or personal relationships that could have appeared to influence the work reported in this paper.

## AUTHOR CONTRIBUTIONS

Hongyao Liu, Xiuli Wu, and Tinghong Ye participated in research design. Hongyao Liu, Xiuli Wu, Cailing Gan, Wei Wei, Xingping Su, Liqun Wang and Lin Yue conducted experiments. Guan Wang contributed to the simulating molecular docking. Hongyao Liu, Xiuli Wu, Qianyu Zhang, Zui Tan and Tinghong Ye performed data analysis. Zhihao Liu, Yuqin Yao, Liang Ouyang and Luoting Yu provided some reagents and consumables. Hongyao Liu and Xiuli Wu contributed to the writing of the manuscript.

## Supporting information

Supplementary Material

## Data Availability

The data used to support the findings of this study are available from the corresponding author upon request.
