# Supplementary material for: A novel multikinase inhibitor SKLB‐YTH‐60 ameliorates inflammation and fibrosis in bleomycin‐induced lung fibrosis mouse models
Source: Cell Prolif. 2021 Jun 14;54(7):e13081. doi: 10.1111/cpr.13081 (PMC8249783; doi:10.1111/cpr.13081)
Supplement: Supplementary file 1 — Supplementary Material [file CPR-54-e13081-s001.doc]

Fig. S1 Synthetic route of YTH-60.

Fig. S2 (A-B)The cytotoxicity of YTH-60 and Nintedanib *in vitro* was confirmed using the normal human liver cell line LO2. (C-D) A549 cells were treated with different concentrations of YTH-60 and Nintedanib for 24, 48 or 72 h and cell viability was measured by MTT. The values are expressed as the mean ± SD (n = 3);*p < 0.05 ; **p < 0.01; *** p < 0.001 compared with YTH-60 control.

Fig. S3 HE staining to evaluate the pathological changes of the heart, liver, spleen, and kidney of each group.

Fig. S1


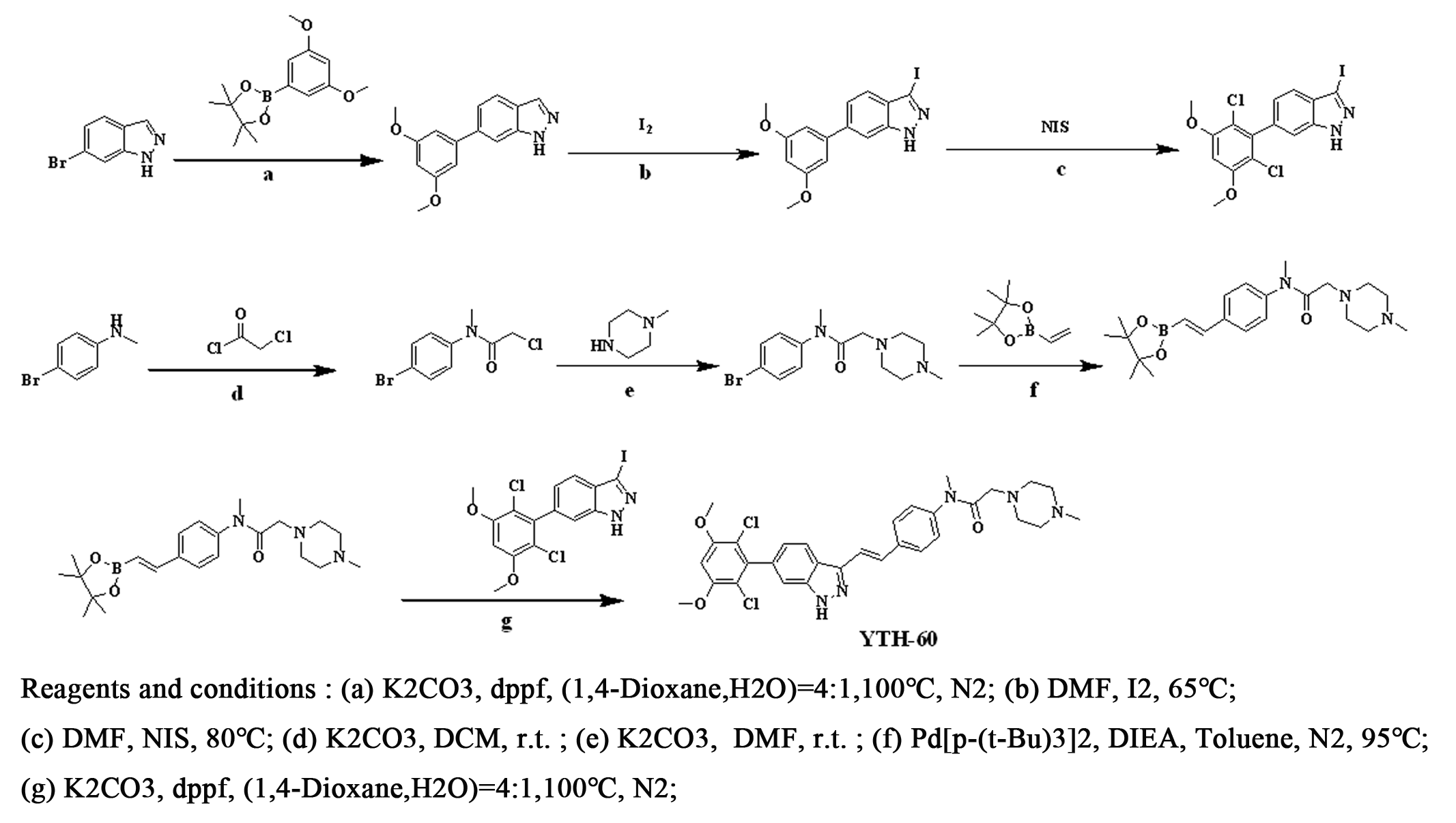


Fig. S2


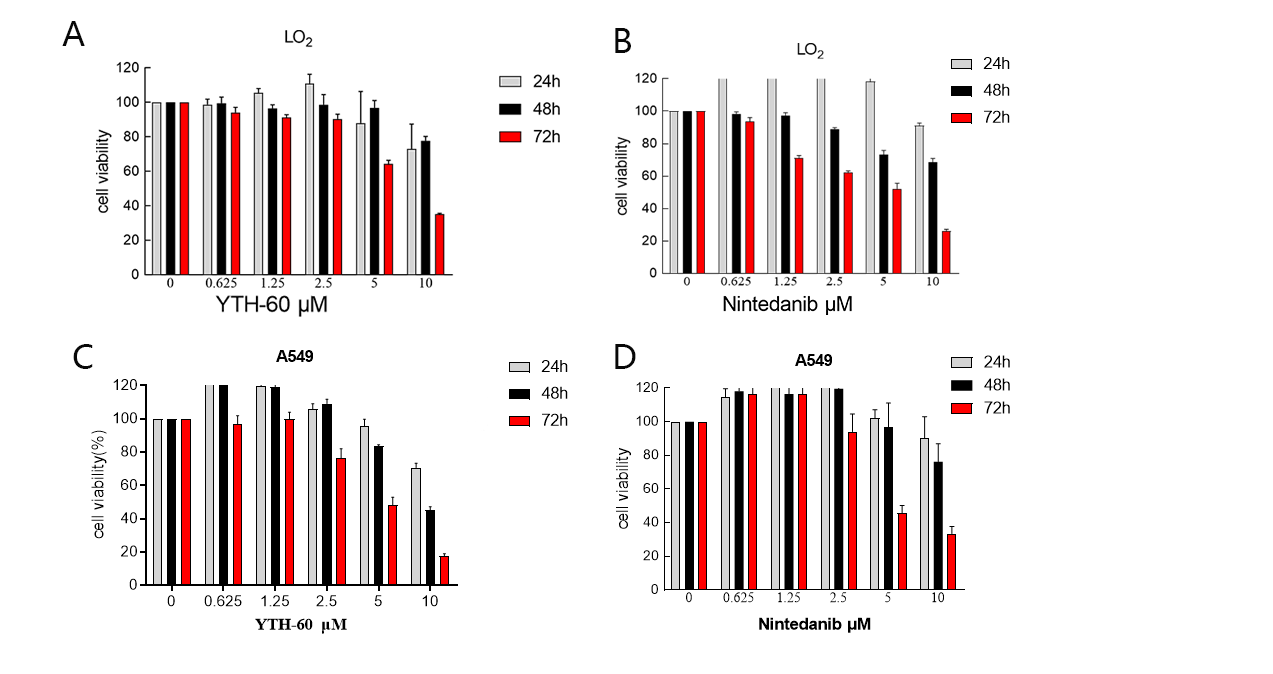


Fig. S3


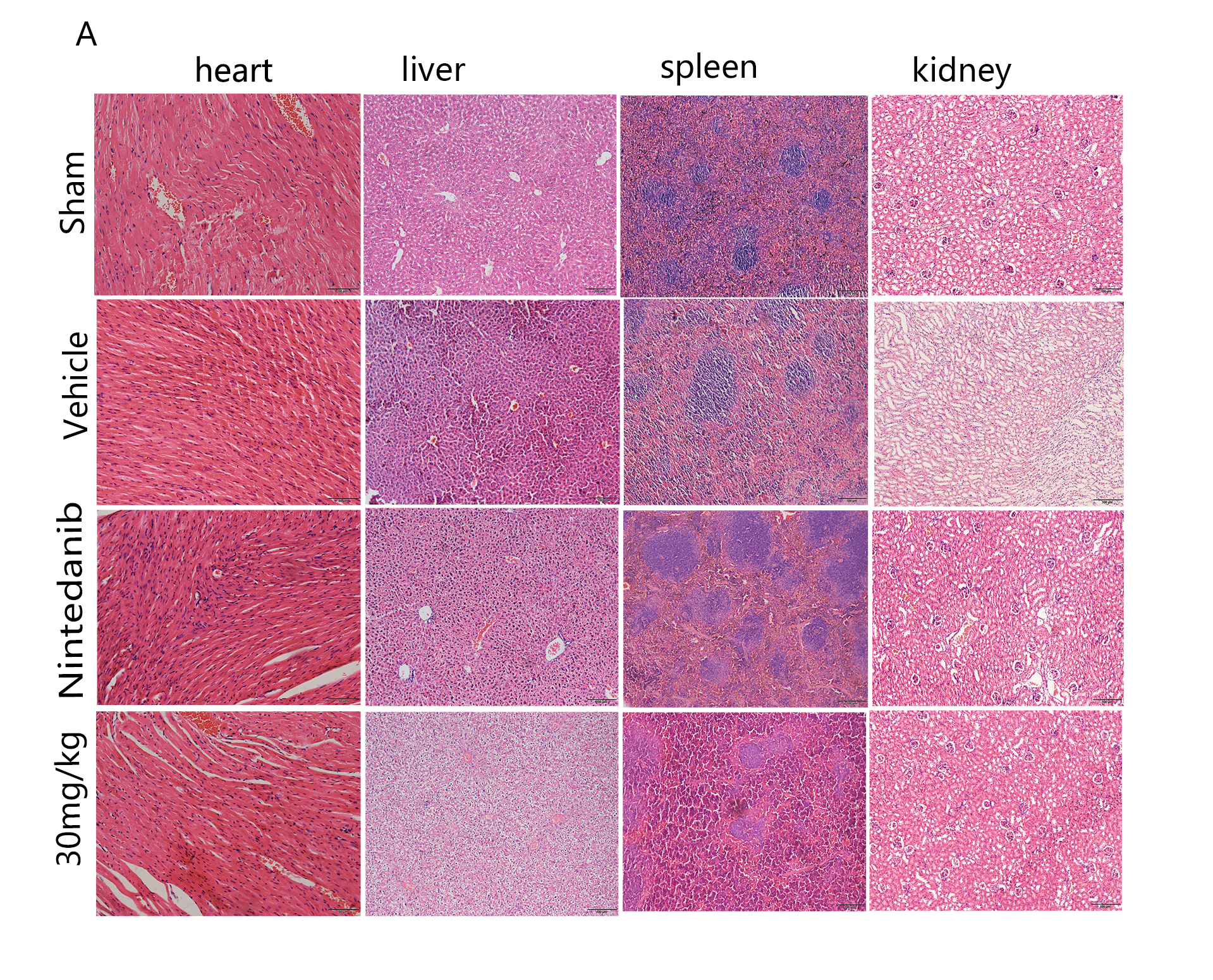


**Table S1** Primer sequences used for real-time qPCR

| **Gene** | **Forward primer(5’-3’)** | **Reverse primer((5’-3’)** |
| --- | --- | --- |
| Mouse α-SMA | CTCTGCCTCTAGCACACAACT | GGCCAGGGCTACAAGTTAAGG |
| Mouse β-actin | GATCTGGCACCACACCTTCT | GGGGTGTTGAAGGTCTCAAA |
| Mouse type I collagen | GGGTGAGACAGGCGAACAAG | AACCAGCAGAGCCAGGGG |
| Mouse TGF-β_1_ | TACGTCAGACATTCGGGAAGCA | AGGTAACGCCAGGAATTGTTGC |
| Human TGF-β_1_ | TGTGTGCTGAAGCCATCGTTG | CCGGCTTGTCTGAAAAGGTCA |
| Human E-cadherin | TGAGAACGAGGCTAACG | TCACATCCAGCACATCC |
| Human vimentin | AAAGTGTGGCTGCCAAGAACCT | ATTTCACGCATCTGGCGTTCCA |
| Human β-actin | CCAACCGCGAGAAGATGA | CCAGAGGCGTACAGGGATAG |

TableS2: Individual and mean plasma concentration-time data of YTH-60 after an IV and PO dose

| IV (2 mg/kg) | | | | | | | PO (20 mg/kg) | | | | | | |
| --- | --- | --- | --- | --- | --- | --- | --- | --- | --- | --- | --- | --- | --- |
| Time Point | Animal ID | | | Mean | SD | CV(%) | Time Point | Animal ID | | | Mean | SD | CV(%) |
| (h) | 101 | 102 | 103 | (ng/mL) | (ng/mL) |  | (h) | 201 | 202 | 203 | (ng/mL) | (ng/mL) |  |
| 0 | No Peak | No Peak | No Peak | NA | NA | NA | 0 | No Peak | No Peak | No Peak | NA | NA | NA |
| 0.083 | 354.99 | 423.84 | 362.43 | 380.42 | 37.79 | 9.93 |  |  |  |  |  |  |  |
| 0.25 | 187.39 | 168.72 | 204.54 | 186.88 | 17.92 | 9.59 | 0.25 | 38.10 | 22.94 | 33.60 | 31.55 | 7.79 | 24.68 |
| 0.5 | 153.36 | 128.91 | 163.29 | 148.52 | 17.69 | 11.91 | 0.5 | 25.31 | 35.39 | 40.62 | 33.78 | 7.78 | 23.04 |
| 1 | 111.92 | 107.71 | 131.39 | 117.00 | 12.63 | 10.80 | 1 | 34.05 | 26.26 | 35.91 | 32.07 | 5.12 | 15.95 |
| 2 | 68.33 | 69.27 | 80.59 | 72.73 | 6.82 | 9.38 | 2 | 35.35 | 57.08 | 87.47 | 59.97 | 26.18 | 43.66 |
| 4 | 41.11 | 42.78 | 48.71 | 44.20 | 4.00 | 9.05 | 4 | 83.13 | 87.88 | 84.72 | 85.24 | 2.42 | 2.84 |
| 6 | 36.84 | 34.45 | 41.43 | 37.57 | 3.55 | 9.44 | 6 | 85.16 | 114.92 | 82.52 | 94.20 | 17.99 | 19.10 |
| 8 | 20.35 | 22.38 | 32.19 | 24.97 | 6.33 | 25.36 | 8 | 79.77 | 97.21 | 67.44 | 81.48 | 14.96 | 18.36 |
| 24 | 2.27 | 2.68 | 3.51 | 2.82 | 0.63 | 22.28 | 24 | 14.36 | 18.49 | 24.52 | 19.12 | 5.11 | 26.70 |

BQL = Below the lower limit of quantitation (LLOQ)
